# Chlorine dioxide flushing protocols for microbial reduction in dental chair units

**DOI:** 10.1371/journal.pone.0342347

**Published:** 2026-03-05

**Authors:** Julian Winkler, Susann Herzog, Felix Dahlhaus, Arndt Matschulat, Helmut Uhlmann, Alexander Mellmann, Thorsten Kuczius

**Affiliations:** 1 Institute of Hygiene, University Hospital Münster, Münster, Germany; 2 a.p.f Aqua System, Wuppertal, Germany; University of Sahiwal, PAKISTAN

## Abstract

Dental chair unit (DCU) waterlines are often microbiologically contaminated. This poses infection risks for patients and dental staff if they are not regularly rinsed and disinfected. This clinical hygiene study evaluated chlorine dioxide (ClO₂) rinsing protocols for microbial and biofilm reduction in DCUs. Automated protocols were tested with varying ClO₂ concentrations and flushing frequencies. Flow cytometry and agar culturing were used to assess microbial load. Continuous low-dose rinsing (1.2 mg/L ClO₂) achieved sustainable microbial reduction (up to 2.51 log₁₀), whereas single high-dose shock disinfections (22.7 mg/L) resulted in transient reductions. ClO₂ was effective in biofilm removal, but its depletion during stagnation highlights the need for continuous application. ClO_2_ seems to be a suitable disinfectant for removing both microbiological contamination and biofilms of DCUs; however, depletion effects of active ClO_2_ were evident underlining the importance of a stable permanent ClO_2_ application. Our results prove that permanent low-dose ClO_2_ application of DCU waterlines is recommended for sustainable water disinfection. A high-concentrated shock disinfection on a periodically basis can be used for biofilm removal, which was demonstrated with experimentally grown biofilm of *P. aeruginosa*.

## Introduction

Dental Chair Units (DCUs) are complex systems along with the dental unit water lines (DUWLs) comprising several meters of water-bearing tubing with small diameters [1]. There are various ways of water suspension into DCUs, either via tap water, via a bottle system or combinations of both [2]. This water is used to rinse and cool dental instruments and to provide the patient´s water for mouth rinsing. As these units are usually operated with non-sterile tap water [3], a high microbiological load can be recorded, especially after long periods of stagnation [4,5]. A case report showed the presence of more than 5,000 coliform bacteria in 100 ml water samples from University Hospital DCUs in Karachi, Pakistan, indicating poor incoming water quality [6]. Nevertheless, cell counts of about 10^5^ cells per mL are described in many cases in the literature, even cell counts of more than 10^8^ cells per mL were found [7–9]. Most of the occurring bacteria are able to form biofilms [10] of which the microbial load is difficult to control in long periods [11]. Even new dental units that have never been in contact with patients showed bacterial contamination [12]. In Germany it is recommended that DCU waters are regularly monitored for total bacterial counts, for the presence of *Legionella* species, and for *Pseudomonas aeruginosa* in samples with increased microbial load [13]. Both types of bacteria belong to pathogenic species, which can cause life-threatening infections, especially in immunocompromised patients. *Legionella* infections can lead to Pontiac fever or to pneumonia known as Legionnaires’ disease [14], which is not always immediately detected due to the low sensitivity of diagnostic tests [15]. Despite antibiotic treatment, Legionnaires’ disease lethality accounts for 13% of clinic-associated cases [16]. *P. aeruginosa* infections can lead to life threatening infections which can be challenging to treat due to its ability of forming biofilms and the lack of antimicrobial agents against it [17].

*Legionella* species and *P. aeruginosa* were detected in several DCU water samples of a dental clinic [2] and dental practices [18]. Patient infections which are clearly linked to the contamination of a DCU are known [19,20]. It should not be ignored that dental staff can be affected as well, by being exposed to aerosols of contaminated water [21]. Hence, the necessity is given to establish an effective disinfection process into a DUWL system that reduces sustainably the bacterial load [22].

Manufacturers of DCUs provide disinfectants that are mainly based on hydrogen peroxide (H_2_O_2_) [23]. Previous experiments have shown that a cell count reduction of about three log_10_ levels of CFU can be achieved with that disinfection [24]. H_2_O_2_ solutions at a high concentration of 2% minimized cell counts sustainable; however, the removal of biofilms took several weeks [25]. DCU water samples became contaminated even after high-dose H_2_O_2_ shock disinfections with *P. aeruginosa* [12] as well as *Legionella* spp. [26] so that H_2_O_2_ should be used as regular dosed disinfectant. Chlorine is another powerful disinfectant with high oxidizing capacity. Its effect is limited to a narrow pH range, by products are [27] and there are depletion effects on in- and organic substances [28].

Several disinfectants are recognized in terms of drinking water hygiene, including chlorine dioxide (ClO_2_) [29]. Another pilot study has already shown that ClO_2_ is more effective than H_2_O_2_ for disinfection in DCUs [30]. ClO_2_ is a disinfectant, which is used in concentrations of up to 2 mg/L in drinking water for its disinfection, odor and taste control [31]. It has higher efficiency than chlorine, especially in a broad pH spectrum, and the use produces less by-products [32]. Other studies have shown that ClO_2_ is also effective against bacterial biofilms and that is able to eliminate viruses [31,33]. It is considered as safe disinfectant with low cytotoxicity [32].

In this study, we tracked rinses according to self-developed flushing protocols, applying ClO_2_ in low doses and as higher-dosed shock disinfection into a DCU for a permanently microbial reduction and biofilm removal. Shock disinfection is primarily intended to reduce high levels of contamination and enlarged biofilms. The use of regular high concentrations of disinfectant is not permitted under regulations and also causes damage to materials. We hypothesize that a continuous low-dose ClO₂ application will maintain microbial reductions more effectively than intermittent high-dose disinfection, due to the limited persistence of ClO₂ under stagnation. The aim was to investigate the efficiency and effects of different flushing protocols using ClO_2_ as a permanent disinfectant.

## Methods and materials

### ClO_2_ disinfection solutions

A ClO_2_ stock solution (0.6% (v/v); Clorious2; a.p.f Aqua System, Wuppertal, Germany) stored at 4°C in the dark, was freshly prepared to the concentration of the working solution that was prepared in tap water (University Dental Clinic Münster) or in filtered tap water using a Baclyser TL filter (Aqua free, Hamburg, Germany). The final ClO_2_ concentrations were adjusted to 1.2 mg/L and 2.4 mg/L respectively, following flushing in individual experimental runs. An initial concentration of 22.7 mg/L in the reservoir was prepared for shock disinfections, so that 15 mg/L ClO_2_ could be determined at the syringe or cup filler after first flushes.

### Determination of the ClO_2_ concentration

The ClO_2_ concentrations were determined using the test device ChlordioXense (Palintest, Gateshead, UK) according to the manufacturer´s instructions. Briefly, a disposable measuring strip was inserted into a 50 mL sample volume while the electrochemical measurement started for 60 seconds followed by indication of the ClO_2_ concentration in mg/L.

### Experimental set up for the rinsing procedures at a DCU and sampling of water probes

**Fig 1:**
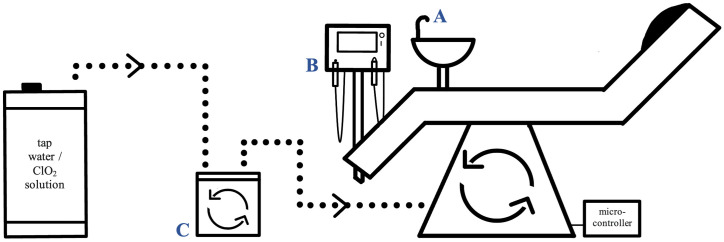
Experimental setup of the dental chair unit used in this study. Rinsing solutions in the barrel (left) were transferred into the chair unit via a pump (C). Automatic flushing of the unit was triggered by a microcontroller (right). Water samples were collected from the cup filler (A), the air/water syringe (B) and the pump (C) as indicated in each case.

In order to monitor the effect of ClO_2_ in this clinical hygiene study on a DCU, a unit (Sirona C4+) was taken out of service for patient care at the University Dental Clinic Münster, Germany to analyze various disinfection rinses with ClO_2_ to find optimized washing programs for sustainable disinfection. Only one DCU was used to follow constant conditions in one system and for good comparability. Using one unit, variations can be avoided in the waterline systems or differences in the initial microbial situation of other DCUs such as various models, ages of the models, other and aged hoses and much more. Dental chairs are rinsed routinely at the beginning and at the end of the working day with municipal tap water, which is free of any disinfections, according to recommendations of the Robert Koch Institute [13]. Between these manually triggered flushes, the tubes are also rinsed using dental instruments on patients. The experimental set-up described here was used to simulate the rinsing and disinfection processes as performed in the dental practice routine (Fig 1). We used a newly developed system for automating the flushing of the DCU.

A barrel with a 30 L capacity contained the washing solutions comprising tap water or tap water mixed with ClO_2_ in concentrations as indicated at the respective experimental runs. To ensure a stable and pressurized water supply (~ 3 bar) to the DCU, a commercially available domestic pressure-boosting pump (Gardena, Ulm, Germany) was employed. The DCU was flushed either with untreated tap water or with ClO_2_ solution as indicated. A Raspberry Pi microcontroller with a custom-developed software (a.p.f Aqua System, Wuppertal, Germany) was implemented to log the actuation of two solenoid valves, which controlled flow for various routine patient-related flushing cycles. Flushing protocols were manually triggered via the DCU control panel, with predefined programs provided by the manufacturer. Hourly flushing over a period of five to seven hours per day was performed to mimic daily clinical work. Water probes were sampled in sterile bottles, containing 20 μg sodium thiosulfate (LP Italiana Spa, Milano, Italy) for inactivation of the ClO_2_ reaction, at the cup filler (A), the air/water syringes (B), and the pump (C) (Fig 1).

### Study design and flushing procedures

**Fig 2:**
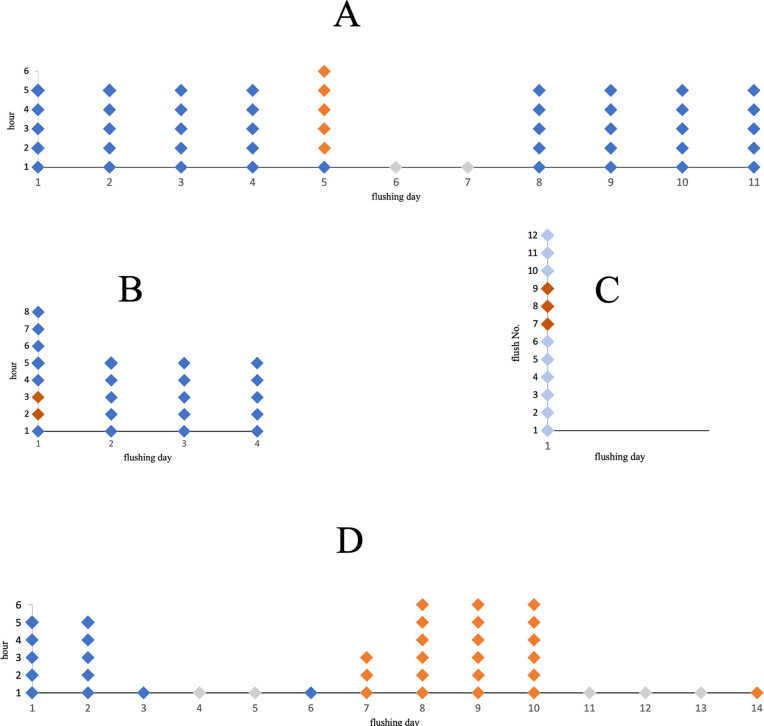
Timeline of flushing protocols for shock disinfection processes and continuous ClO_2_ applications. The figure shows the course of the individual flushing protocols and the respective disinfection measures, whereby the individual phases including stagnation are intended to reflect a real working situation in a practice. On the days indicated (flushing day), a DCU was flushed several times at hourly intervals (hour) or in a row. Each individual rhombus in the illustration corresponds to a flushing process. The flushing processes were carried out with either tap water (dark blue rhombus) or filtrated tap water (light blue rhombus). Disinfections were performed with the low ClO2 concentrations of 1.2 mg/L (A, D) and 2.4 mg/L (D), as indicated in the individual test approaches (orange rhombus), and shock disinfections are depicted as dark orange rhombus (B, C). Days of stagnation without flushing are marked as grey rhombus.

The following rinsing programs were carried out to test the effect of single and multiple treatments with ClO_2_ solutions using concentrations as indicated regarding efficient microbial reduction (Fig 2). Two aspects were considered in particular: the effect of shock disinfection procedures with limited ClO_2_ flush runs (Fig 2 B and C) and the effect of repeated rinsing with ClO_2_ in concentrations used in the range according to the German drinking water ordinance (Fig 2 A and D).

### ClO_2_ shock disinfection procedures

To follow cell reduction in the contaminated DCU, shock disinfection protocols were checked for efficient and sustainable disinfection.

After three days of stagnation and subsequent rinsing with tap water (blue rhombus; each blue rhombus represents a single tap water rinse), the unit was rinsed once with ClO_2_ for shock disinfection and a second time after 60 minutes exposure time (Fig 2B). The concentration amounted to at least 15 mg/L ClO_2_ in the dental unit measured at the outlet of the syringe or cup filler. The subsequent rinses were carried out with tap water over the following days.

Following the effect of shock disinfection based on a lower planktonic bacterial load, rinsing with filtered tap water (transparent blue rhombus) was carried out to minimize the primary addition of a natural bacterial load from tap water. After a three-day stagnation period, six flushes in a row followed with filtered tap water (transparent blue rhombus). Shock ClO_2_ disinfection flushes (orange rhombus) were carried out three times followed by rinsing with filtered tap water (Fig 2C).

### Flushing procedures with ClO_2_

Each flushing protocol started with tap water rinsing (blue rhombus, Fig 2D) for standardization and to track the initial contamination rate in the DCU. The symbols represent the respective flushing process, whereby a one-hour interval was maintained between the individual flushes so that a maximum of six flushes were carried out in one day. After stagnation for two days (day four and five; grey rhombus) a single tap water flushing occurred on day six followed by disinfection flushes with ClO_2_ (orange rhombus). To recognize the impact of multiple ClO_2_-treatments on a sufficient disinfection, flushing programs were implemented day-by-day starting with 1.2 mg/L and 2.4 mg/L ClO_2_ solutions in tap water, respectively. Stagnation for three days followed a final disinfection flush.

After flushing processes with tap water (each blue rhombus represents a single tap water rinse) for four days, the disinfection day started with a single tap water flush followed by five flushes with 1.2 mg/L ClO_2_ (orange rhombus). After a stagnation period (grey rhombus), flushes with tap water followed as indicated for the following four days (Fig 2A).

### Microbiological determination from rinsed water samples by cultivation

DCU water probes were sampled after defined flushing steps as indicated. For detection of cultivable microorganisms after treatment with ClO_2_, reactions were stopped by sampling water probes in sterile polypropylene disposable cups containing 20 μg sodium thiosulfate (LP Italiana Spa, Milano, Italy). Aliquots of 500 µl volumes were directly plated on R2A agar plates (Roth, Karlsruhe, Germany) for cultivation of bacteria. Plates were incubated at 36°C for 48–96 h and colony-forming units (CFU) were counted and calculated to CFUs per mL.

### Total cell counting and live-dead-determination of microorganisms by flow cytometry

The flow cytometry (FCM) technique was used for analyzing the total bacterial cell counts (TCC) in the water samples from the DCU. The FCM technique performed on a NovoCyte Flow Cytometer 2000R (ACEA Biosciences Inc., USA) was used for tracking microbiological membrane integrity (intact cell counts, ICC) and dis-integrity (disrupted cell counts, DCC) after the exposure to ClO_2._ For the microbial live-dead determination (ICC vs. DCC), aliquots of 100 µl were transferred into a 96-well microtiter plate. The dyes SYBR Green I (Invitrogen, Thermo Fisher Scientific, Massachusetts, USA) and propidium iodide (PI; Roth, Karlsruhe, Germany) were used for membrane integrity and dis-integrity staining, respectively. Stock solutions of the fluorescence dyes were suspended in dimethyl sulfoxide (DMSO; Roth, Karlsruhe, Germany), whereby SYBR Green I 10.000 X stock solution diluted 1:100 was used, and PI was added at a final concentration of 6 µM. For homogenous mixing in the wells, microtiter plates were agitated shortly at 1.000 rpm on a horizontal shaker (Bioer, Hangzhou, China) and incubated at 38°C for exactly 12 min in the dark. Samples were finally analysed at a volume of 50 µL with a flow rate of 35 µL/min. A laser at a wavelength of 488 nm was used for excitation and fluorescence signals were detected with photomultipliers at 520 ± 20 nm in the green light spectrum and at 630 nm in the red-light spectrum. In between the samples, the flow cytometer was rinsed with 200 µL sterile filtered, deionized water, omitting the dyes to determine the intrinsic fluorescence and background matrix signals. The light signals were converted to electronic signals and were finally obtained as logarithmic data. The characteristic gates were adjusted to the signal clusters based on the flow cytometer manufacturer and to initial measurements of untreated bacteria resuspended in tab water. Data were analysed using the NovoExpress 1.3.0 software and a gating procedure as described [34]. Background noise by cell debris and ions was separated for the quantification by gating. No compensation was used for the assays.

Data resulted from 50 µl volumes were calculated to values per mL. The total cell count (TCC) consisted of the sum of ICCs and DCCs and was calculated by using the formula:


ICC (intact  cell  counts)/ [50 μl + DCC (dead  cell  counts)/ [50  μl]) * 20

### Impact of ClO_2_ on bacterial biofilms

The reference strain *P. aeruginosa* (ATCC 27853), originating from American Type Culture Collection (ATCC) and purchased from the German Collection of Microorganisms and Cell Cultures (Leibniz Institute DSMZ, Braunschweig, Germany) was used as a biofilm model. The impact of ClO_2_ on microbiological biofilms was analyzed. Colonies of *P. aeruginosa* were grown overnight at 36°C on Columbia blood agar plates (Oxoid, Wesel, Germany). Cells, suspended in yeast medium consisting of 0.5% (w/v) yeast extract (Roth, Karlsruhe, Germany) and 1% (w/v) glucose in 0.85% (w/v) saline solution, were adjusted to 10^8^ cell/ml. Volumes of 150 µl were transferred into 96-well plates with U-bottom (Greiner Bio-One, Frickenhausen, Germany) and incubated at room temperature for 48 h for biofilm formation. For biofilm staining supernatants consisting of planktonic cells were discarded and biofilms were carefully washed twice with 10 mM PBS (phosphate buffered saline; Merck, Darmstadt; Roth, Karlsruhe, Germany). A ClO_2_ solution, at a concentration of 15 mg/L prepared in PBS, layered the bacterial biofilm for 60 min. This step was repeated once as the reaction was finally stopped with 0.1 mol/L sodium thiosulfate (Merck, Darmstadt, Germany). After discarding the supernatant, biofilm was stained with 0.1% (w/v) crystal violet solution (Merck, Darmstadt, Germany) for 20 min. Unbound colour solution was removed by washing with PBS. The intensity of the blue colouration, which indicated the presence of biofilm, was visually documented.

### ClO_2_ depletion effects by different types of water

The presence of in- and organic substances in a watery matrix and the properties of materials within the chair unit, e.g., synthetic hoses, may have a relevant impact on depletion of ClO_2_. To analyze such depletion effects, the ClO_2_ concentration was determined over time after the flushing processes. ClO_2_ in distilled water with 1.2 mg/L rinsed the unit six times in one approach. The second series was carried out by five disinfection steps (2.4 mg/L ClO_2_) followed by six purges with 1.2 mg/L ClO_2_ in distilled water as well. The rinsing processes were carried out at intervals of one hour at which samples were taken at the pump (C) and at the cup filler (A) (Fig 1) followed by electrochemical ClO_2_ concentration measurement as described. As control, 70 µL of the ClO_2_ stock solution was prepared in a glass bottle with 1 L sterile filtered and deionized tap water (Milli-Q, Merck, Darmstadt, Germany) to follow the ClO_2_ depletion by the dilution matrix over time. In the next experimental set-up, the ClO_2_-depletion effect of the watery matrix linked to the presence of in- and organic ingredients was investigated. Some selected physicochemical parameters of tap water of the Institute of Hygiene, Münster, and deionized water were analyzed, such as pH, conductivity as well as calcium, magnesium, iron, and total organic carbons (TOC) in accordance to DIN EN ISO 38404, EN 27888 as well as DIN EN ISO 7980, DIN EN ISO 38406, and DIN EN 1484, respectively. Sterilization occurred by autoclaving at 121°C for 20 min and filtration through filters (0.2 µm pore size; Filtropur V25, Sarstedt, Nümbrecht, Germany). Analyzing the impact of organic loads, bacteria were artificially added to the tap water matrix. For this purpose, *Escherichia coli* bacteria (ATCC 25922, obtained from the German Collection of Microorganisms and Cell Cultures (Leibniz Institute DSMZ, Braunschweig, Germany)) were used at a final concentration of 10^5^ cells/ml. The bacterial solution was added either alive or inactivated by heat (95°C, 10 min). The ClO_2_ concentrations in the assay solutions were determined hourly using the Palintest.

### Analysis of data

Each individual sample was measured three times in the flow cytometer, and the respective intact cell counts, cell counts with membrane damage and total cell counts were determined using the NovoExpress 1.3.0. software. Values were determined as triplicates and the respective mean values were calculated from the individual results using Microsoft Excel software, and the corresponding standard deviation was determined. In order to recognize the clear effect of ClO_2_ and to be able to verify sufficient cell count reduction and possible disinfection, the cell count was converted into log_10_ units by Excel.

## Results

### Chlorine dioxide and biofilm degradation

ClO_2_ is valued as a potent oxidizing agent usable over a wide pH range and has been used successfully for bacterial reduction and disinfection in various applications [35–37]. Even in aqueous or watery environments, exposure to ClO_2_ resulted in a significant cell reduction of hygienically relevant microorganisms living in the planktonic state [38].

**Fig 3:**
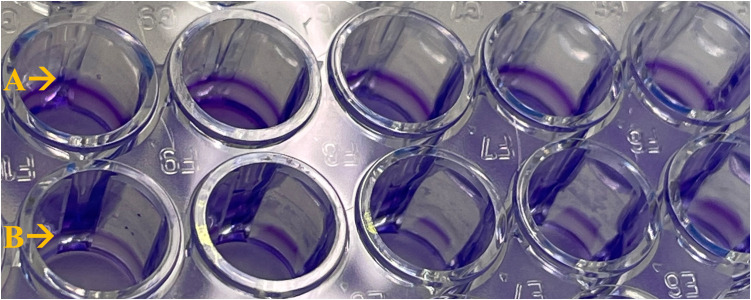
Reduction of P. aeruginosa biofilms by ClO_2_. Biofilm cells of P. aeruginosa, cultured in a 96-well plate for two days, were treated twice with 15 mg/L ClO2 in PBS for 60 min. The reaction was stopped with sodium thiosulfate. Biofilms were stained with crystal violet afterwards. ClO2 treated wells (row B) showed clearly less crystal violet staining, revealing the impact of ClO2 on biofilms through visible gaps and reduced bandwidth compared to untreated wells (row A).

As microorganisms form biofilms in water-bearing systems of DCUs, we investigated the impact of ClO_2_ in reducing and dissolving biofilms. *P. aeruginosa* was chosen as a model organism for biofilm formation. However, other waterborne bacteria are capable of forming biofilms as well [39,40]. Artificially produced biofilms of *P. aeruginosa* in a 96-well plate degraded visibly, demonstrated by crystal violet staining after two shock disinfection procedures. Complete dissolvement of biofilms or large gaps in biofilm structures were detected (Fig 3).

### Repeated ClO_2_ flushes in a one-day disinfection and rinsing protocol

A DCU was rinsed with a ClO_2_ solution from a connected reservoir at defined intervals using a pump system controlled by a microcontroller. The volume was 1 L in each case with a flushing time of approximately 2 min. The total bacterial load in the DCU was determined using the flow cytometry technique, whereby all bacteria were recorded, including those that cannot be cultivated under routine conditions.

First, a one-day ClO_2_ rinsing period with both low ClO_2_ concentrations and a shock disinfection followed by repeated flushes with tap water and with filtered tap water was investigated to ensure a possible sustainable reduction in the bacterial content.

Repeated rinsing processes started with tap water for four days, as indicated (Fig 2A). Samples from either the syringe or the cup filler were analysed using FCM (Table 1).

**Table 1:** Bacterial reduction of one-day ClO_2_ treatment following tap water flushes.

Day	Flush after hours as indicated	ClO_2_^*^ (mg/L)	Syringe		Cup filler	
			ICC^**^ (log_10_)	ΔICC^**^ (log_10_)	ICC^**^ (log_10_)	ΔICC^**^ (log_10_)
**1**	1	0	5.65		6.21	
	5	0	5.65	0.00	5.54	0.67
**2**	1	0	5.76		5.99	
	5	0	5.46	0.30	5.59	0.40
**3**	1	0	5.75		5.99	
	5	0	5.38	0.37	5.35	0.64
**4**	1	0	5.70		5.92	
	5	0	5.32	0.38	5.33	0.59
**5**	1	0	5.53		5.86	
	2	0	5.20	0.33	5.61	0.25
	3	0.14	4.27	1.26	4.94	0.92
	4	0.07	3.73	1.80	4.75	1.11
	5	0.12	4.12	1.41	5.00	0.86
	6	0	3.90	1.63	4.70	1.16
**8**	1	0	5.75		5.71	
	5	0	5.14	0.61	5.14	0.57
**9**	1	0	5.68		5.58	
	5	0	5.31	0.37	5.46	0.12
**10**	1	0	5.65		5.78	
	5	0	5.13	0.52	5.19	0.59
**11**	1	0	5.65		5.69	
	5	0	5.25	0.40	5.36	0.33

* On day 5, the initial concentration of the ClO_2_ solution in the barrel was 1.2 mg/L leading to the actual measured concentrations mentioned above. The concentrations indicated are the ClO_2_ means concentrations measured in the syringe/ cup filler of the DCU in triplicates.

** ICC = intact cell count, ΔICC = decrease in ICC values starting from the first value of each flushing day.

The values presented are means of three replicates.

Intact cell counts (ICC) and changes from the initial ICC of each flushing day (ΔICC) were calculated alongside measured ClO_2_ concentrations. An ICC-number decrease, indicating cell damage, after ClO_2_ application corresponded to a strong effect of a disinfectant. The five daily rinses with tap water at hourly intervals reduced the bacteria content by a maximum of 0.38 log_10_ levels in the syringe and by 0.67 log_10_ levels in the cup filler (Table 1). Regular rinsing resulted in a slight reduction in microorganisms indicated through falling ICC values. There was no massive decrease in microbiology due to the numerous rinsing processes with tap water. However, this result was expected, as the addition of further tap water also flushed new waterborne bacteria into the system.

After the processes and a final tap water flush, ClO_2_ in an initial solution of 1.2 mg/L rinsed through the DCU (Fig 2A) for five times at hourly intervals at day five. Regular ClO_2_ rinsing resulted in a more pronounced cell count reduction with a maximum of 1.8 log_10_ and 1.16 log_10_ levels in syringe and cup filler, respectively, compared to initial bacterial load. Thus, regular and repeated ClO_2_ flushing resulted in a sustained lower intact cell count of 3.73 log_10_ in the syringe and 4.7 log_10_ in the cup filler compared to initial values of 5–6 log_10_. Numbers of intact cells fall while the total cell load, including the membrane-damaged bacteria, decreased compared to the initial situation. However, this means that rinsing with ClO_2_ caused an effect with membrane damage. The ClO_2_ concentration was photometrically detected after the second rinse, because the value of the first rinse was below the detection limit, and it is supposed that ClO_2_ was depleted from surfaces as well as inorganic and organic materials. The effectively measured concentrations in the DCU were a maximum of 0.14 mg/L.

After following a two-day stagnation period, the DCU was repeatedly rinsed with tap water without adding disinfectant (starting at day 8). Interestingly, the bacterial load was as high as at day one with an ICC of 5.75 log_10_/ 5.71 log_10_ of this experimental set-up after the last ClO_2_ flush. Again, the repeated flushing resulted in a daily cell count decrease of maximal 0.61 log_10_ states in both syringes and cup fillers.

### Two step shock ClO_2_ disinfection followed by tap water flushes

As multiple rinsing with low ClO_2_ doses in a one-day-period did not lead to a sustainable reduction in microorganisms, the use of shock disinfection was tested as impact for a possible sustainable reduction. After an initial rinse with tap water, a single shock disinfection with initial 22.7 mg/L ClO_2_ in the barrel was carried out. A second ClO_2_ flush after a one-hour exposure time followed by rinsing with tap water for five times in a row and over a further period of three days at hourly intervals (Fig 2B and Table 2). Samples from either the syringe or the cup filler were again analysed by FCM. The bacterial content was differentiated in total cell counts and intact cell counts. Changes from the initial ICC (ΔICC) were calculated. Based on an intact cell count of 6.66 log_10_ and 6.54 log_10_ in syringe and cup filler, respectively, the shock disinfection resulted in a maximum ICC reduction by 2.54 log_10_ levels in syringe and up to 2.44 log_10_ levels in cup filler within two days.

**Table 2:** Single ClO_2_ shock disinfection and tap water flushes.

Day	Flush number as indicated	Syringe		Cup filler	
		ICC^*^ (log_10_)	ΔICC^**^ (log_10_)	ICC^*^ (log_10_)	ΔICC^**^ (log_10_)
**1**	1	6.66		6.54	
	3	4.22	2.45	5.35	1.19
	8	4.13	2.54	4.25	2.28
**2**	1	4.14	2.52	4.10	2.43
	5	4.50	2.16	4.65	1.89
**3**	1	4.47	2.20	4.37	2.17
	5	4.81	1.86	4.71	1.83
**4**	1	5.10	1.56	5.13	1.40
	5	5.18	1.48	5.26	1.27

The initial concentration of the ClO_2_ solution in the barrel was 22.7 mg/L.

* ICC = intact cell count ** ΔICC = decrease in ICC values starting from the first value of the first flushing day. The values presented are means of three replicates.

The two-step ClO_2_ shock disinfection showed an effect after 1 h exposure time. The intact cell count decreased by 2.45 log_10_ and 1.19 log_10_ in syringe and cup filler, respectively. After further rinses with tap water, the ICC in syringe reduced by further 0.09 log_10_ levels and remained almost constantly low at about 4.1 log_10_ for at least 24 h, despite stagnation over several hours and further subsequent rinses. In the following with five daily rinses and stagnation, the ICC increased continuously and achieved a value of 5.18 log_10_ after 72 h. The TCC remained consistently high (about 5.5 log_10_) over this entire period after shock disinfection, considering fluctuations due to variable cell counts in the incoming tap water.

A similar progression of ICC and TCC counts was seen with a slight delay in the cup filler. The maximum ICC cell count reduction was achieved after 24 hours. Here, the ICC increased during the stagnation and rinsing periods, and it reached nearly the initial value after ClO_2_ treatment again after 72 hours (5.26 log_10_). The TCC also remained consistently high in the cup filler as past shock disinfection.

### Three-step shock ClO_2_ disinfection followed by flushes with filtered tap water

Even if the cell number was below the initial cell count, there was a steady increase in the ICC. We tested whether the bacterial reduction efficiency can be sustainably reduced by ClO_2_ treatment with a low added bacterial load. The effect of a ClO_2_ shock disinfection in the dental chair unit was investigated with the entry of a lower bacterial content using filtered tap water for the use in the barrel. The DCU was initially flushed six times in a row with filtered water (Fig 2C). Initial ICCs were determined with 6.13 log_10_ cells in syringe and with 6.40 log_10_ cells in cup filler. The DCU was rinsed with ClO_2_ solution (14.1 mg/L measured concentration in the DCU) three times at hourly intervals (flush number 7–9 as indicated in Fig 2C). The TCC and the ICC were determined alongside measured ClO_2_ concentrations and differences to the initial ICC and TCC values (Table 3).

The ICC decreased by about 1 log_10_ level to 3.40 log_10_ and 3.61 log_10_ in syringe and cup filler, respectively. In contrast, TCC increased to 5.08 log_10_ and 5.20 log_10_ in syringe and cup filler, respectively. This indicated that biofilm cells being present in the system were washed out and were damaged by the disinfectant.

**Table 3:** ClO_2_ shock disinfection and flushes with filtered water.

Flush number as indicated	ClO_2_^*^ (mg/L)	Syringe				Cup filler			
		ICC^**^ (log_10_)	ΔICC^**^ (log_10_)	TCC^***^ (log_10_)	ΔTCC^***^ (log_10_)	ICC^**^ (log_10_)	ΔICC^**^ (log_10_)	TCC^***^ (log_10_)	ΔTCC^***^ (log_10_)
**1**	0	6.13		6.18		6.40		6.44	
**6**	0	4.46		4.59		4.48		4.66	
**9**	14.1	3.40	1.06	5.08	0.49	3.61	0.87	5.20	0.54
**12**	0	3.10	1.36	4.17	0.42	3.40	1.08	4.35	0.31

* The initial concentration of the ClO_2_ solution in the barrel was 22.7 mg/L at flush 7. The respective average concentration measured is mentioned in the table.

** Intact cell count (ICC) and decrease in ICC values starting from the 6^th^ flush (ΔICC).

*** Total cell count (TCC) and decrease in TCC values starting from the 6^th^ flush (ΔTCC).

The values presented are means of three replicates.

Subsequently, the system was rinsed three times in a row with filtered water. The ICC in the two samples decreased again slightly and the TCC decreased after the last rinse as well.

A one-day repeatedly rinsing period with disinfectant and a single shock disinfection resulted in bacterial reduction but proved to be insufficient enough for sustainable bacterial reduction in the DCU. Therefore, continuous rinsing periods were carried out using the disinfectant. Materials in contact with the ClO_2_ solution showed no signs of visible damage or discoloration.

### Continuous rinsing processes

Prior to disinfection, the DCU was characterized by flushing with tap water and stagnation (Fig 2D). ClO_2_ at concentrations of either 1.2 or 2.4 mg/L were used as initial disinfection solutions (Fig 2D). Flushing started on day seven and rinsing occurred for the following four days. Samples from either the syringe or the cup filler were analysed by FCM. Based on the cell counts prior to the first measurable ClO_2_, the intact cell count reduction (ΔICC) after the individual rinses was determined (Table 4).

The baseline intact cell count was 5.71 log_10_ in syringe and 6.26 log_10_ in cup filler prior to rinsing with 1.2 mg/L ClO_2_. Cell counts were at 6.12 log_10_ and 6.35 log_10_, in syringe and cup filler, respectively, before rinsing with 2.4 mg/L ClO_2_.

**Table 4:** Repeated rinsing protocols using different concentrations of ClO_2._

Day	Flush after hours as indicated	1.2 mg ClO_2_			2.4 mg ClO_2_		
		ClO_2_^*^ (mg/L)	ΔICC^**^ (log_10_)		ClO_2_^*^ (mg/L)	ΔICC^**^ (log_10_)	
			Syringe	Cup filler		Syringe	Cup filler
**6**		0			0		
**7**	1	0.34	0.18	0.87	0	0.16	0.22
	2	0	1.12	1.06	0.48	0.75	1.80
	3	0.04	1.81	1.52	0.33	2.12	2.04
**8**	1	0	1.30	1.45	0	1.71	1.89
	2	0	1.18	1.55	0.09	2.08	1.46
	3	0.21	1.56	1.83	0.45	2.00	2.01
	4	0.16	1.72	1.97	0.28	2.12	2.24
	5	0.14	1.79	2.01	0.44	2.28	2.24
	6	0.09	2.05	2.51	0.38	2.55	2.58
**9**	1	0	0.85	1.66	0	1.77	2.10
	2	0	1.31	1.76	0	2.46	2.23
	3	0.27	1.66	1.89	0.55	2.08	2.10
	4	0.16	1.76	2.01	0.53	2.26	2.14
	5	0.16	2.15	2.31	0.49	2.31	2.28
	6	0.09	1.84	2.09	0.33	2.38	2.31
**10**	1	0	1.01	1.65	0	1.86	1.98
	2	0	1.40	1.81	0.03	2.33	1.68
	3	0.15	1.64	1.90	0.56	2.14	2.14
	4	0.17	1.76	2.02	0.49	2.27	2.22
	5	0.15	1.83	2.07	0.27	2.37	2.43
	6	0.10	2.28	2.47	0.41	2.43	2.45
**14**	1	0	−0.49	0.57	0	0.81	−0.01

* The initial concentration of the ClO_2_ solution in the barrel was 1.2 mg/L and 2.4 mg/L, respectively, the respective concentration measured is mentioned in the table starting on day 6.

** Intact cell count (ICC) and decrease in ICC average values starting from the last flush on day 6 flush (ΔICC). The values presented are means of three replicates.

When using 1.2 mg/L ClO_2_, the cell count decreased by 1.81 log_10_ levels in syringe and by 1.52 log_10_ levels in the cup filler after three rinses. On the following rinsing day (day eight) with freshly prepared ClO_2_, the cell count decreased by 2.05 log_10_ and 2.51 log_10_, on day nine by a maximum of 2.15 log_10_ and 2.31 log_10_ and on day ten by a maximum of 2.28 log_10_ and 2.47 log_10_ levels in syringe and cup filler samples, respectively. Stagnation followed up to day 14 prior to a final ClO_2_ flush. Cells recovered up to this day (Table 4).

**Fig 4:**
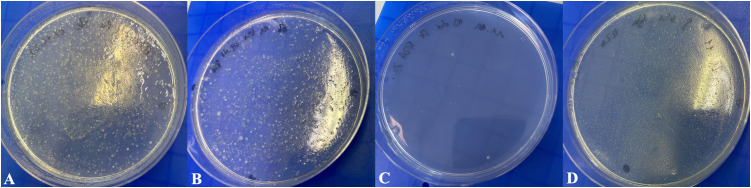
Plating results on R2A agar with continuous ClO_2_ application. According to the flushing protocol by using 2.4 mg/L ClO2 water samples taken from the syringe were plated in 500 µl volumes on solid R2A medium followed by incubation at 36°C for 96h. Cultivatable bacteria multiplied as visible cfu. The plates represent bacterial lawn formation after stagnation (day 1, hour 1; A), reduced lawn formation on day two after six flushing hours; B), few and scattered bacteria after treatment with 2.4 mg/L ClO2 (day ten, hour six) and recovering after stagnation (day 14; hour 1 D).

In addition to FCM, we have also carried out the classic method of agar cultivation for visual inspection. Water samples taken at four different times in the experimental set-up with use of 2.4 mg/L ClO_2_ according to Fig 2D were plated on solid R2A media. Bacterial cultivation of the stagnation flush with tap water (sample from day 1) showed lawn formation (Fig 4A). Additional five rinsing steps on day two resulted in a slightly less bacterial lawn (Fig 4B) while low growth was achieved on day 10 (flush after hour 6; Fig 4C), which match the results of the FCM (Table 4 and Fig 4). These cultivation results corresponded the FCM results. On day 14, after the first flush, bacterial lawn formation appeared again.

The microbial killing efficiency of the 2.4 mg/L ClO_2_ solution was similar to 1.2 mg/L (Table 4). The cell count was reduced by a maximum of 2.55 log_10_ levels in syringes and up to 2.58 log_10_ levels in cup filler during the rinses over the days.

**Fig 5:**
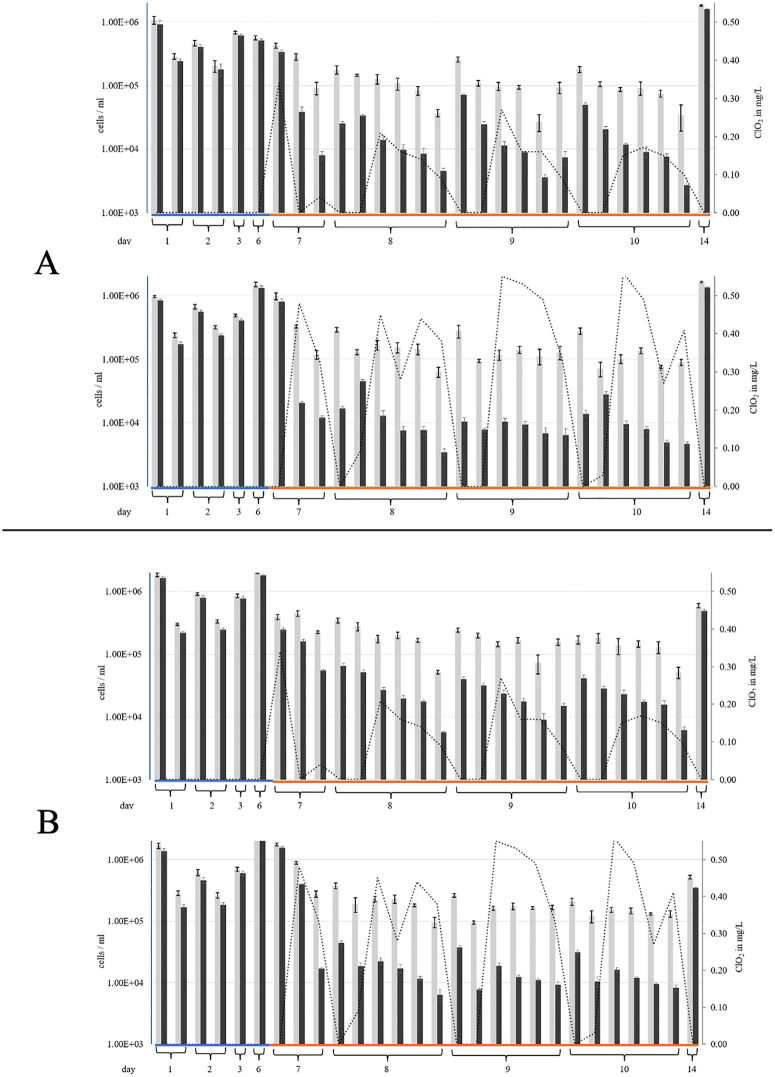
Correlation of the total cell counts (TCC), the intact cell counts (ICC) and the ClO_2_ concentration during the study period. According to the flushing protocol (Fig 2D), the DCU was flushed at indicated days either with tap water (blue line) or with ClO2 solutions (orange line) at initial concentrations of 1.2 mg/L (A) and 2.4 mg/L (B). Total cell counts (TCC; grey columns) and intact cell counts (ICC; black columns) were calculated alongside the ClO2 concentrations (dotted line), determined using the Palintest, either from the syringe (upper diagram) and from the cup filler (lower diagram). Each column (mean value ± standard deviation) represents the flushing day and hour according to Table 4.

It turned out that the ClO_2_ concentration showed clearly lower values than the initial solution on the one hand and on the other a continuous decrease of the ClO_2_ concentration after repeated rinses despite fluctuations what indicated depletion effects (Fig 5 and Table 4).

Fig 5 shows the absolute numbers of the total cell counts (TCC) and the intact cell counts (ICC) after rinsing with tap water and ClO_2_ solutions. Both, the TCC and the ICC, remained constantly high at approximately 10^5^ to 10^6^ cells/ mL during the rinses with tap water. Starting from day seven, ClO_2_ solution was introduced at concentrations of 1.2 mg/L (Fig 5, upper diagrams) and 2.4 mg/L (Fig 5, lower diagrams), and the concentrations were determined during the experiment. Samples were analysed from the syringe and from the cup filler. After the second rinse, the ICC decreased and dropped sharply after the third rinse to 7.95 x 10^3^ mL^-1^ cells measured in the syringe with 1.2 mg/L ClO_2_ and 1.2 x 10^4^ mL^-1^ cells with 2.4 ClO_2_ mg/L. This ICC number remained low, even when fluctuations were taken into account, and it continued to decrease with further flushing processes. Lowest ICC counts were measured in the syringe on day 10 with 1.2 mg/L. The ICC was at 2.71 x 10^3^ mL^-1^ cells on the last flush of the day while the effective measured ClO_2_ concentration was 0.10 mg/L at this moment. Lowest ICC count achieved with 2.4 mg/L was 3.45 x 10^3^ mL^-1^ cells measured on day eight with the last flush also in the syringe. The measured ClO_2_ concentration was 0.38 mg/L. After stagnation without any rinsing processes, bacteria partially recovered, which was reflected to an increase in ICCs in the first determinations on days eight, nine and ten. It was noticeable that the ClO_2_ concentration decreased during the stagnation phase. Due to the low ClO_2_ concentration, bacteria were able to recover. After prolonged stagnation (day 14), the ICC content was again high close to the initial situation of days one to six with values up to 1.59 x 10^6^ mL^-1^ cells measured in the syringe.

### Depletion and impairments for consideration of using ClO_2_ for rinsing DCUs

**Fig 6:**
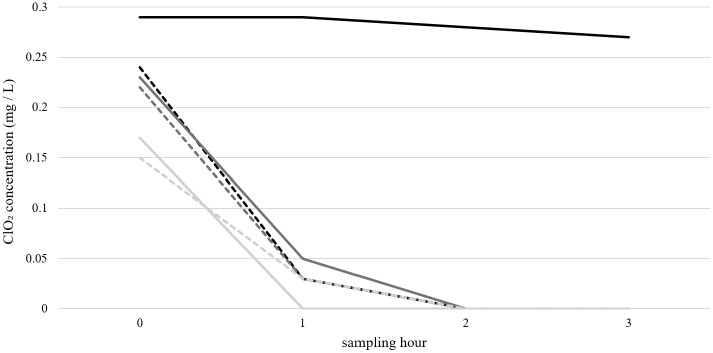
ClO_2_ depletion effects in various types of water.

In a previous study, we showed that depletion of ClO_2_ in solutions comprising organic substances, e.g., presence of bacteria in tap water, should not be underestimated [38]. To test the impact of depletion in liquids used as dilution matrices of the ClO_2_ stock solution, an initial concentration of 0.4 mg/L ClO_2_ was added to various types of water. The decrease of the ClO_2_ disinfectant was monitored over a three-hour period using the Palintest (Fig 6).

The ClO_2_ concentration was measured in mg/L directly after adding ClO_2_ in an hourly manner for three hours in total. The measurement was performed as a single determination.

Starting with ClO_2_ concentrations of 0.15 to 0.24 mg/L in the respective samples, ClO_2_ concentration decreased very rapidly to < 0.05 mg/L within the first minute. In case of deionized and sterile water, values decreased only by 0.02 mg/L starting from 0.29 mg/L.

The depletion effect in in-house tap water with a natural live bacterial load was comparatively analysed in sterile-filtered tap water and in autoclaved tap water. These water types are characterized by the presence of specific organic and inorganic substances, such as proteins, lipids and DNA of bacteria. Some representative physicochemical parameters of in-house tap water compared with sterile filtered, deionized water are presented in S1 Table.

Having similar organic loads due to the pre-treatments, these water types showed a rapid and continuous decrease in ClO_2_ concentrations. After two hours, no active ClO_2_ was detectable. Interestingly, a comparable rate of decrease was observed after the addition of 5 log_10_ E. coli cells to the sample, regardless of whether bacterial cells were alive or killed by autoclaving.

**Fig 7:**
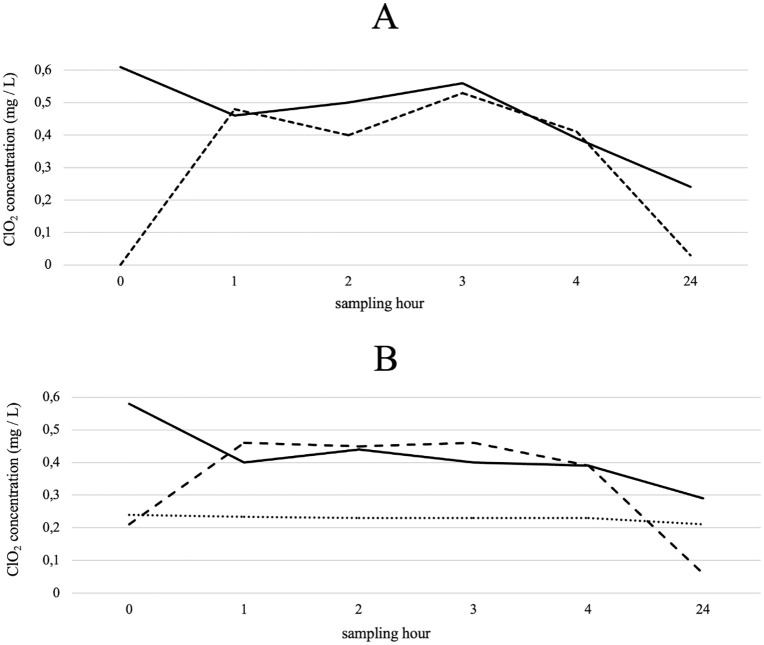
Analyses of the ClO_2_ depletion effects for supply line and final use. The DCU was flushed hourly, as indicated, and after 24 h with aqueous ClO2 solutions of initial 0.58 mg/L ClO2 (A) and 0.61 mg/L ClO2 (B). The concentrations were directly measured in mg/L with the Palintest device at the pump outflow (black line) and after passing the DCU at the final collection point (cup filler) (dashed line). A ClO2 solution prepared in a sterile glass bottle served as control (dotted line).

Next, we analysed the depletion effect in the DCU under real conditions with a natural higher bacterial content due to stagnation (Fig 7A) and further, with a very low bacterial content achieved by the application of 2.4 mg/L ClO_2_ prior to the analysis (Fig 7B). In both test approaches, ClO_2_ concentration was added to distilled water in the barrel. Probing the pump, concentrations of 0.58 mg/L (Fig 7A) and 0.61 mg/L ClO_2_ (Fig 7B) were detectable. Furthermore, the ClO_2_ concentration at the cup filler was determined after the rinsing solution had flushed the whole DCU. Within the first hour, the concentration of ClO_2_ at the pump site decreased in both test approaches to 0.48 mg/L (A) and 0.46 mg/L (B), respectively. The concentrations in the DCU achieved 0.46 mg/L (A) and 0.4 mg/L (B) ClO_2_. The concentrations stagnated over the following hours, as the values decreased overnight. The control suspension in a glass bottle remained constant with ClO_2_ concentrations between 0.21 mg/L and 0.24 mg/L.

## Discussion

Dental unit waterlines are often supplied with local tap water consisting of natural heterotrophic waterborne microorganisms that colonize and form biofilms [41]. To keep the bacterial content sufficiently low and to minimize the risk of infection of patients and staff, extensive flushing of DCU with disinfectants is recommended before and after each working day as well as after each patient treatment [13]. Although regular disinfection is still based on hydrogen peroxide products [42] and chlorine [43], an alternative approach could be the use of the oxidative disinfectant ClO_2._ ClO_2_ is active in a wide pH range, forms less amounts of by-products, has high disinfection efficiency to microorganisms [38] and is efficient to resolve biofilms. Biofilm detachment was proven through our experiments with highly concentrated ClO_2_ acting for short periods.

We investigated the efficiency of the oxidative disinfectant ClO_2_ as an appropriate disinfectant for rinsing the DCU to minimize the microbiological colonization on the one hand. On the other hand, we tracked bacterial reduction and recolonization by using various rinsing processes and protocols. The aim was to find a suitable protocol for disinfections with ClO_2_ that would result in a sustained reduction of microorganisms and minimize the disinfectant concentrations added. The rinsing protocols depicted a symbolic daily clinical routine with several phases of use and standstill throughout the day, followed by multiple days of tap water stagnation in the system.

Our experimental setup used a closed system of tap water and disinfectant using one DCU model in order to minimize artificial effects resulting from other models, other water lines and further additional impacts from outside. In this case we had a stable system for testing. The ClO_2_ concentrate was mixed with tap water via an established micro-controlled system to obtain a stable and measurable ClO_2_ concentration flushing into the DCU. Using an automated disinfection unit, the ClO_2_ solution was added to the incoming tap water via a dosing system up to an effective concentration measurement. The total cell counts and the membrane-damaged bacteria were determined using the flow cytometry technique (FCM) that enables the detection of all microorganisms being present. This method can be used to determine cell counts fast and sensitive and to differentiate between viable and dead cells [44,45]. Another advantage of the FCM technique is the potential to identify viable but now cultivable cells (“VBNCs”) [46].

We found that the intact cell counts in the DCU reached up to 10^6^ cells/mL after a few days of stagnation. First, we used a protocol for flushing with a low ClO_2_ concentration for only one day. The ICC decreased more than just using tap water for flushing, but higher reductions than 1.8 log_10_ levels with ICC counts between 3.73 log_10_ and 4.7 log_10_ were not achieved under these conditions. Beyond that, the cell counts quickly recovered up to 5.75 log_10_ when omitting ClO_2_. Interestingly, the cell counts of the cup filler were higher than in the syringe. A reason for that could be a higher throughput of water from the syringe compared to the cup filler [41]. However, the extent of this effect remains to be investigated, because opposite effects were overserved in other studies as well [26,47].

Bacterial loads were reduced more relevant with a high-concentrated ClO_2_ shock disinfection protocol whereby an ICC reduction of up to 2.54 log_10_ was possible resulting in cell counts of 4.13 log_10_ ICC. However, here as well, the cell counts quickly recovered to nearly initial values. To analyze a sustainable reduction of bacterial loads, we flushed the DCU with filtered tap water and used a high ClO_2_ concentration for disinfection. ICC values reduced from up 4.46 log_10_ in the syringe and 4.48 in the cup filler to 3.10 log_10_ and 3.40 log_10_, respectively. The numbers of TCCs came up by about 0.5 log_10_ because of removal of biofilm cells. The results highlight that ClO_2_ is an effective agent for decreasing cell counts in the DCU and that biofilm can be reduced visually and measurably without material change and damage. No material damage was observed in experiments involving low-dose ClO_2_. The long-term material compatibility under prolonged exposure or higher ClO_2_ concentrations requires further studies.

In another approach, we permanently applied 1.2 mg/L ClO_2_ over a period of several days to the DCU water. We measured increased reductions of the ICCs of up to 2.51 log_10_ from the baseline cell count of 6.26 log_10_ compared to the single day ClO_2_ flushing protocol. A log_10_ reduction of 2.51 corresponds to approximately 99.7% inactivation of microorganisms. This bacterial reduction, shown by flow cytometry results, was comprehensible to the cultivation method whereby the latter method was not as sensitive. However, cultivation is considered as the standard method, as all reduction units refer to this method. Plating samples from stagnation water, bacteria formed lawns on the agar plate whereas a maximum of only 20 CFUs were visible after rinsing the DCU with 2.4 mg/L ClO_2_. The continuous application demonstrated an increased disinfection effect with higher sustainability than a single shock disinfection, as well as using the solution only for one day. This method complied with permission of residual ClO_2_ concentrations of 0.2 mg/L after water treatment according to the Germany´s central environmental authority [48]. ClO_2_ does not appear to be harmful in such concentrations, as no toxicity in a subchronic oral toxicity test was found with up to 40 ppm ClO_2_ in drinking water [49]. A permanent ClO_2_ application of 2.4 mg/L did not result in relevant higher cell count reductions, therefore half of this concentration is sufficient for disinfection.

During the experiments, it became apparent that the measured ClO_2_ concentration in the DCU decreased over several hours regardless of the presence of high or low cell counts. This effect was more recognizable in the DCU itself than in the pump. For comparison, the concentration curve was recorded in a glass bottle with remaining constant and stable concentrations. This is due to depletion caused by reactions with in- and organic substances present in the water [50,51]. Another factor for ClO_2_ depletion is the polymer tubing in the DCU [52], since the surface area of the tubing in the unit is the greatest [53–55]. These depletion effects underline the importance of constant supply of active ClO_2_ solutions into the DCU when developing an automated disinfection unit to stabilize the disinfectant concentration. On the other hand, the permanent ClO_2_ flow may have a negative impact on the longevity of the DCUs tubing [56]. ClO_2_ has been less widely used than other disinfections such as H_2_O_2_. ClO_2_ was considered more unstable and complicated for use before stable ClO_2_ products came on the market [30]. H_2_O_2_ is considered effective for use in DCUs in relation to planktonic contamination and biofilms [25]. However, there are already studies conducted on DCUs that proof ClO_2_ to be more effective compared to alkaline hydrogen peroxide [57]. ClO_2_ is considered to be more effective than H_2_O_2_ in terms of biofilm removal [30], whereby in our experimental set-up we only wanted to consider the removal of the biofilm without other possible impacts such as shear stress or interactions with the tube material. Besides that, no microbial resistance to ClO_2_ is documented [58].

As shown, other studies also present good results with other oxidatively effective disinfectants. The effect resulting in a reduction in cell count is primarily based on the cultivation method. Our series of investigations are based on a cultivation-independent methodology that detects all microorganisms, including those that cannot be cultivated. Rinsing with tap water introduces new bacteria that are detected by the FCM technique but cannot be determined using the cultivation-based technique. The effect shown here is therefore species-independent and cannot be compared with cultivation-based studies.

Finally, based on results of our experiments, it can be concluded that ClO_2_ is a suitable disinfectant for DCUs from a microbiological point of view. The investigations include a possible application of ClO_2_ as an alternative disinfection method as a snapshot. However, we are aware that the investigations do not allow for a generally valid statement, as the investigations are limited in that only one DCU was examined, a real-world patient use simulation was omitted, the biofilm test procedure was simplified, and no long-term test procedures were carried out. However, in application-related systems, we recommend adding freshly prepared ClO_2_ permanently in low doses (approximately 1.2 mg/L) to the tap water in order to achieve a sustainable disinfection effect and to avoid depletion effects. Shock disinfections may reduce and inactivate bacteria and biofilms when DCUs are already and highly contaminated after a long-term standstill with tap water stagnation. Similar results have been reported as ClO_2_ was an effective disinfectant to low cell counts in DCU waterlines when using the increased concentration of 20 mg ClO_2_/L [33] and can effectively decontaminate biofilms [59].

## Conclusion

ClO_2_ seems to be a suitable disinfectant that removes both microbiological contamination and biofilms from the DCU. Permanent low-dose ClO₂ flushing (1.2 mg/L) is recommended to sustainably reduce microbial contamination in dental chair units. Periodic high-dose shock treatments (22.7 mg/L) may be reserved for biofilm removal. Continuous application is essential to counteract ClO₂ depletion and ensure long-term disinfection efficacy.

## Supporting information

S1 Table Physicochemical parameters of in-house tap water and sterile filtered, deionized water (MilliQ-water). (DOCX)
