# Supplementary material for: Chlorine dioxide flushing protocols for microbial reduction in dental chair units
Source: PLoS One. 2026 Mar 5;21(3):e0342347. doi: 10.1371/journal.pone.0342347 (PMC12962484; doi:10.1371/journal.pone.0342347)
Supplement: S1 Table — (DOCX) [file pone.0342347.s001.docx]

# **Supporting information**

**S1 Table: Physicochemical parameters of in-house tap water and sterile filtered, deionized water (MilliQ-water)**

| **Parameters** | **Tap water** | **MilliQ-water** |
| --- | --- | --- |
| **pH** | 7.60 | 6.11 |
| **Conductivity (µS/cm)** | 494 | 0.7 |
| **Calcium (mg/L)** | 68.6 | < 0.5 |
| **Magnesium (mg/L)** | 4.5 | < 0.1 |
| **Iron (mg/L)** | < 0.05 | < 0.05 |
| **TOC (mg/L)** | 4.2 | 0.0 |
